# Vancomycin-Stabilized Copper Nanoclusters: Isolation by Solid-Phase Extraction and Application in Chiral Capillary Electrophoresis

**DOI:** 10.3390/nano16211361

**Published:** 2026-10-26

**Authors:** Ekaterina A. Kolobova, Andrei I. Demenshin, Yulia P. Zimarina, Elena V. Solovyeva

**Affiliations:** Chemistry Institute, Saint-Petersburg State University, Universitetskaya nab. 7/9, 199034 Saint Petersburg, Russia

**Keywords:** metal nanocluster, capillary electrophoresis, chiral selector, enantioselectivity, solid-phase extraction, amlodipine, acenocoumarol

## Abstract

Utilizing state-of-the-art nanomaterials for chiral discrimination is an emerging area of analytical research. Herein, we explored for the first time, metal nanoclusters as a chiral selector in capillary electrophoresis. Vancomycin-stabilized copper nanoclusters (CuVanc NCs) were intentionally synthesized, purified and characterized by UV-Vis and fluorescence spectroscopy, transmission electron microscopy, dynamic light scattering, and X-ray photoelectron spectroscopy. The 5-step sequential protocol of solid-phase extraction was designed to isolate CuVanc NCs from the as-synthesized solution before electrophoretic experiments. The recovery was found to be over 90%. The purified CuVanc NCs were utilized as a chiral discriminator for enantioselective recognition of model drugs. The effects of key parameters, including the pH value of the running buffer and concentration of CuVanc NCs, were assessed. Chiral separation was successfully achieved for both positively and negatively charged analytes, with the better enantioselectivity observed for amlodipine (*α* = 1.017) and acenocoumarol (*α* = 1.015), respectively. The separation behavior was strongly influenced by the background electrolyte pH and nanocluster concentration, balancing electrostatic interactions and electrophoretic mobility. Overall, the study highlights a high potential of metal nanoclusters as effective chiral selectors in capillary electrophoresis, opening up new opportunities for chiral analysis and pharmaceutical quality control.

## 1. Introduction

Chirality is a vital matter in modern analytical, pharmaceutical, and biomedical chemistry, as enantiomers of the same molecule can exhibit different, sometimes even opposite, biological activity, toxicity, and pharmacological profiles. Therefore, reliable separation and quantification of enantiomers is essential for the development of safe drugs, enantiomeric purity control, pharmacokinetic and toxicological studies, and the analysis of food, environmental, and agrochemical samples [1,2]. The real-world significance of this task is further emphasized by the fact that more than half of the drugs used in therapy are chiral, while only about a quarter are used as an individual enantiomer, and racemic forms may retain a less active or even side-effecting component [3].

Capillary electrophoresis (CE) has become a powerful tool of enantioseparation due to high efficiency, low sample and selector consumption, rapid analysis, and flexibility of separation regimes [4,5,6]. An additional advantage of CE is that a chiral selector can be used easily by simple addition to the background electrolyte, allowing for variation in the selector’s nature, concentration, pH, and medium composition, quickly screening different recognition systems. Furthermore, CE allows one to control the order of enantiomer migration and exploits differences in the electrophoretic mobility of analyte-selector complexes, which not only improve separation but also help to elucidate a mechanism of chiral recognition [7].

Despite the widespread use of cyclodextrins and other traditional chiral selectors [8,9,10,11], the search for new effective selectors remains one of the most pressing challenges in chiral CE, as no universal selector exists and satisfactory separation of many compounds is not achieved by conventional approaches. This challenge stimulated the active introduction of functional materials into chiral CE based on ionic liquids [12,13,14,15], deep eutectic solvents [16,17,18,19], nanoparticles [20,21,22,23], metal–organic frameworks [24,25,26] as modifiers, pseudo stationary phases, and new selectors.

Against this background, nanomaterials occupy a special place because they combine size effects, chemical and mechanical stability, large specific surface area, and ease of surface modification [27]. This is critically important for chiral analysis because the modified nanostructures can enhance enantioselective interactions, act as carriers of traditional selectors, and provide new recognition regimes at the interface. It has already been shown that gold nanoparticles [28,29,30,31], magnetic iron oxide nanoparticles [32], carbon nanotubes, and mesoporous silica nanoparticles [33,34] can be successfully joined with chiral selectors to perform electrokinetic chromatography and capillary electrochromatography systems. Metal nanoclusters, a relatively new class of nanomaterials, appear to be also promising for determination of enantiomers, but they have been probed only in optical analysis methods [35,36,37].

Metal nanoclusters are very small particles consisting of a countable number of atoms and having a size up to 3 nm [38,39]. This structural feature distinguishes the nanoclusters from metal nanoparticles and ensures their similarity with molecular objects. Due to quantization of electronic states, metal nanoclusters exhibit molecular properties such as luminescence, molecular magnetism, and other qualities [40,41]. Modern applications of metal nanoclusters include fluorescent sensing [42,43,44,45,46,47] and diagnostics, antibacterial treatment and therapy [48,49,50], and chemical catalysis [51,52]. Due to simple variation in stabilizing ligands (small or large molecules like amino acids, peptides, polymers, DNA), metal nanoclusters are promising for chiral separation in capillary electrophoresis. Their shell becomes not a passive component but a key part of the selector, determining hydrogen bonds, inclusion in hydrophobic cavities, and electrostatic and aromatic interactions, on which the enantioseparation depends.

It is vital to underscore that the current use of metal nanoclusters as modifiers of electrophoretic systems is extremely limited. Our previous study is the only example available to the best of our knowledge. In this study, it was demonstrated that L-proline-stabilized copper nanoclusters can serve as a pseudo-stationary phase, which alters the electrophoretic migration parameters of serotonin reuptake inhibitor drugs [53]. In the present study, relying on the developed approach, we aimed to expand the application of metal nanoclusters in CE for chiral separation. For this purpose, a glycopeptide antibiotic vancomycin was selected as a stabilizing agent of copper nanoclusters. Vancomycin is a natural compound with multiple stereogenic centers and its different functional groups capable of mediating enantioselective interactions, which include hydrogen bonds, hydrophobic interactions, and the participation of aromatic moieties and amide groups [54]. This is why vancomycin has long established itself as an effective chiral selector in CE: it has been used to separate over 100 racemates, achieving high resolution values for many compounds [54,55,56,57].

Since a chiral selector is usually introduced directly into the background electrolyte in CE, its purity is important. This is abundantly essential for proof-of-concept studies, which testify to new selectors, including nanomaterials. Therefore, the isolation and purification of vancomycin-stabilized copper nanoclusters (CuVanc NCs) after the synthesis were a separate significant task of the study. Despite the widespread use of metal nanoclusters in fluorescence spectroscopy and catalysis, their separation techniques are limited by laborious and high solvent consumption methods, which are poorly compatible with the task of obtaining concentrated purified solutions [58]. In this context, solid-phase extraction (SPE) appears to be suitable, as it relies on rapid and selective sorption processes, allows for customization of the sorbent surface, and is compatible with preconcentration. Despite SPE having already proven the effectiveness in the related task of the nanomaterials field, like a separation of metal nanoparticles from a complex matrix [59,60], its application for metal nanoclusters is less prevalent. Therefore, an original protocol of SPE purification of vancomycin-stabilized copper nanoclusters was developed at the first stage of the study before their use in chiral CE.

## 2. Materials and Methods

### 2.1. Materials and Chemicals

Copper sulfate (BioChem, Karlsruhe, Germany), vancomycin (lyophilisate from Krasfarma, Moscow, Russia), sodium hydroxide (Reakhim, Saint-Petersburg, Russia), hydrochloric acid (Reakhim, Saint-Petersburg, Russia), boric acid (Reakhim, Saint-Petersburg, Russia), methanol (Fluka, Buchs, Switzerland), N,N-dimethylformamide (DMF) (Sigma, Darmstadt, Germany), disodium ethylenediaminetetraacetic acid (EDTA) (Sigma, Darmstadt, Germany), sodium dihydrogen phosphate dihydrate (Reakhim, Saint-Petersburg, Russia), cetyltrimethylammonium bromide (CTAB) (Sigma, Darmstadt, Germany), phosphoric acid (Reakhim, Saint-Petersburg, Russia), acetic acid (Reakhim, Saint-Petersburg, Russia), sodium acetate trihydrate (Sigma, Darmstadt, Germany).

All aqueous solutions were prepared on high-purity deionized water with a resistivity of 18 MΩ cm (Millipore, Molsheim, France).

### 2.2. Synthesis of Vancomycin-Stabilized Copper CuVanc Nanoclusters

The synthesis of vancomycin-stabilized copper nanoclusters was carried out without the use of an external reducing agent according to the following procedure. To 10 mL of aqueous vancomycin solution (50 mg/mL) 61 μL of a 1.14 M CuSO4 solution was added under vigorous stirring. The mixture was stirred for 5 min and then incubated in a thermostat at 80 °C for 72 h. Then, the mixture was cooled to room temperature and centrifuged for 20 min at 12,140 *g*. The resulting white precipitate was removed and the collected supernatant represented a final solution of CuVanc NCs.

### 2.3. Isolation of CuVanc Nanoclusters

To isolate CuVanc NCs from the as-synthesized solution, a solid-phase extraction (SPE) protocol was developed, which included the following steps. SPE tube Strata C-18T (55 um, 140 Å) 500 mg/3 mL (Phenomenex, Torrance, CA, USA) was initially preconditioned with 2 mL acetonitrile twice, followed by equilibration with 2 mL of methanol and 2 mL of water acidified with formic acid (0.1% *v*/*v*). 1 mL of crude CuVanc NCs solution was added to an SPE column and flushed with 2 mL of 20% MeOH acidified with formic acid (0.1% *v*/*v*). Then, CuVanc NCs were eluted with 50% MeOH acidified with formic acid (0.1% *v*/*v*). Subsequently, the tube was washed with 2 mL MeOH acidified with formic acid (0.1% *v*/*v*) twice. The collected fraction of CuVanc NCs was concentrated 3–4 times by evaporation at 80 °C. The obtained solution was used for the electrophoretic experiments.

### 2.4. Characterization of CuVanc Nanoclusters

#### 2.4.1. Molecular Spectroscopy

Absorption spectra of CuVanc NCs were recorded in a quartz cuvette with 1 cm optical path length using a UV-1800 spectrophotometer (Shimadzu, Kyoto, Japan). Deionized water was used as a reference sample. Fluorescence spectra were acquired on a FluoroMax spectrometer (Horiba Jobin Yvon, Paris, France), with exit and entrance slit widths of 2 nm and an integration time of 1 s.

#### 2.4.2. High-Performance Liquid Chromatography

For analysis of copper nanoclusters by high-performance liquid chromatography with fluorescence and diode-array detection (HPLC-FlD-DAD), a Shimadzu liquid chromatograph (Kyoto, Japan) was used under the following conditions: a Phenomenex Luna column with a stationary phase of octadecylsilane C18, geometric dimensions of 150 mm × 2.1 mm, and a pore size of 100 Å. Mobile phase A: water acidified with 0.1% formic acid (*v*/*v*); mobile phase B: methanol acidified with 0.1% formic acid (*v*/*v*); the flow rate of the mobile phase was 0.3 mL/min. The separation was carried out in the gradient elution mode: starting from 10% phase B, increasing the concentration of phase B from 10 to 15% over 0–3 min, holding for 5 min at 15% phase B, then increasing the concentration of phase B from 15 to 35% over 8–13 min, holding from 13 to 16 min at 35% phase B, then increasing the concentration of phase B from 35 to 100% over 16–19 min, holding from 19 to 22 min at 100% phase B and returning to the initial conditions in 0.1 min, followed by conditioning the system for 5 min.

The HPLC-DAD method for the determination of vancomycin was partially validated in accordance with current analytical guidelines [61,62] by assessing linearity, limit of quantification, detection limit, intraday and interday precision, and intraday and interday accuracy (Supplementary Materials, Figure S1, Table S1). The method exhibited a linear response within the range of 0.005–1 mM for vancomycine (r^2^ > 0.9995), with limits of detection/limits of quantification of 0.0008/0.0025 mM.

Determination of CuVanc NCs was performed using HPLC-FlD. Separation conditions were the same as previously described. Since there is no possibility of precise quantification for CuVanc NCs, only the linearity and repeatability of peak area for different dilution factors of nanocluster solution after SPE were assessed within a validation. The validation parameters of relative CuVanc NCs concentration are given in Supplementary Materials (Table S2, Figure S2).

#### 2.4.3. High-Resolution Transmission Electron Microscopy

High-resolution transmission electron microscopy (HRTEM) was performed using a Libra 200FE electron microscope (Carl Zeiss, Jena, Germany) with an accelerating voltage of 200 kV. HRTEM images were recorded in scanning mode from three random areas of the sample. In order to prepare the sample for HRTEM, a solution of CuVanc NCs was dropwise applied to the surface of a carbon film, after which the substrate was gently blown with a blower to evaporate the water.

#### 2.4.4. Dynamic Light Scattering

Dynamic light scattering (DLS) analysis of CuVanc NCs solutions was carried out on a Zetasizer Nano ZS analyzer (Malvern Instruments Ltd., Great Malvern, UK). The refractive index value of 0.24 was used for copper in the Smoluchowski equation. The viscosity of the solvent was set to 0.887 cP for water and the refractive index was set to 1.33. Zeta potentials were determined using the same instrument in a microelectrophoresis regime.

#### 2.4.5. X-Ray Photoelectron Spectroscopy

A Thermo Fisher Scientific Escalab 250Xi spectrometer (East Grinstead, UK) using monochromatic Al Kα radiation was used to collect the X-ray photoelectron spectra (XPS). The analyzer pass energies for survey and high-resolution spectra were set to 100 eV and 50 eV, respectively. The position of the carbon C1s peak was assumed to be 284.8 eV and used as an internal standard to determine the binding energy of other photoelectron peaks. In order to prepare samples for XPS measurements, 5 μL of CuVanc NCs solution was drop-cast on a silicon slide and air-dried.

#### 2.4.6. Fourier-Transform Infrared Spectroscopy

Fourier-transform infrared (FTIR) spectra were measured on an IRAffinity spectrometer (Shimadzu, Kyoto, Japan). The measurements were performed in the attenuated total reflectance regime with dry samples in a diamond cell at 25 °C. The resolution was set at 4 cm^−1^, the number of scans was 50, and the wavenumber range was 750–4000 cm^−1^.

### 2.5. Capillary Electrophoresis Measurements

Capillary electrophoresis was performed using a KAPEL^®^-205 capillary electrophoresis system (Lumex, Saint-Petersburg, Russia) with spectrophotometric detection (deuterium lamp, spectral range from 190 to 380 nm) in hydrodynamic sample injection mode. Quartz capillaries with a total length of 60 cm and an effective length of 52 cm, with an internal diameter of 50 μm and external polyimide coating, were used in the experiments. Electrophoretic data was processed using Elforan-205 software, version 4.2.5 (Lumex, Saint-Petersburg, Russia).

A new bare fused-silica capillary was preconditioned by sequential rinsing with dimethylsulfoxide for 10 min at 2000 mbar, deionized water for 10 min at 1000 mbar, 0.1 M hydrochloric acid for 10 min at 1000 mbar, deionized water for 5 min at 1000 mbar, 0.5 M sodium hydroxide for 15 min at 2000 mbar, and deionized water again for 10 min at 1000 mbar. Electroosmotic flow (t_m_ = 3.85 ± 0.15 min) was evaluated using 10 mM borate buffer (pH 9.3) as a background electrolyte; after that, the capillary was rinsed with deionized water for 10 min.

In the electrophoretic experiments, a sodium phosphate background electrolyte in the pH range of 2–10 was employed. pH value was adjusted to the required value using concentrated phosphoric acid or 4 M sodium hydroxide.

For quantitative analysis of Cu^2+^, a background electrolyte consisted of 1 mM CTAB, 8 mM EDTA, acetate buffer solution 10 mM and pH 5.5. The sample injection was of 10 s × 50 mbar, and the detection wavelength was of 225 nm. The calibration curve was constructed in the concentration range 0.1–2.5 mM. Partial method validation was performed for the following parameters: linearity, limit of quantification, detection limit, intraday and interday precision, and intraday and interday accuracy. Average intraday and interday migration time was also assessed. All information is presented in the Supplementary Materials (Figure S3, Table S3).

Electrophoretic experiments for chiral separation were performed in 5–100 mM phosphate background electrolyte at pH 6–8 with the addition of vancomycine (0.5 and 1 mM) or CuVanc NCs solution diluted 5–50 times.

The analyzed solution consists of a racemic mixture of acenocoumarol (100 μg/mL), ketoprofen, flurbiprofen, and amlodipine (25 μg/mL). Sample injection was hydrodynamic during 5 s at 50 mbar; the detection wavelength was 255 nm.

The enantioselectivity (*α*) was calculated using the formula:

(1) $$\alpha =\frac{{\vec{\mu }}_{i}-{\vec{\mu }}_{\mathrm{EOF}}}{{\vec{\mu }}_{j}-{\vec{\mu }}_{\mathrm{EOF}}},$$

in which electrophoretic mobilities were calculated as follows:

(2) $${\vec{\mu }}_{i}=\frac{L_{\mathrm{tot}}\cdot L_{\mathrm{eff}}}{U\cdot t_{i}}$$

${\vec{\mu }}_{i,j}$—total mobility of analyte (one of the enantiomers) or EOF, ${\vec{\mu }}_{\mathrm{EOF}}$—electroosmotic flow mobility, *L_tot_* and *L_eff_*—total and effective lengths of capillary (cm), *U*—applied voltage (V), *t_i_*—migration time of enantiomers or EOF marker (s).

Resolution (*R_s_*) was calculated using the formula:

(3) $$R_{s}=\frac{2\cdot (t_{j}-t_{i})}{W_{i}+W_{j}}$$

*t_i,j_*—migration time of enantiomers, *W_i,j_*—the enantiomer peak width at base.

Efficiency (*N*, theoretical plate numbers (t.p.) was automatically calculated using the data processing software of the instrument system.

## 3. Results and Discussions

### 3.1. Isolation, Purification, and Characterization of CuVanc Nanoclusters

Formation of metal nanoclusters relies on grouping a few to hundreds of metal atoms into tiny nanostructures which are stabilized by organic ligands. A simplified scheme of the formation of copper nanoclusters in the presence of vancomycin is shown in Figure 1a. The ultrasmall size of metal nanoclusters splits continuous energy bands into discrete, molecule-like energy levels which ensure their peculiar optical properties. The key characteristic of transition metal nanoclusters is luminescence, which serves as a primary criterion of successful synthesis. Accordingly, absorption and fluorescence spectra were recorded first for the as-synthesized CuVanc NCs solution. These spectra are shown in Figure 1 in comparison with the spectra of vancomycin to demonstrate the distinctive properties of the nanoclusters. As can be seen, CuVanc NCs exhibit a new absorption band at 355 nm and intense fluorescence in the range of 420–470 nm at the corresponding excitation wavelength.

The obtained crude CuVanc NCs solution was analyzed by HPLC with fluorimetric and diode-array detection. The choice of mobile phase was limited since the nanoclusters are poorly soluble in acetonitrile; therefore, the analysis was carried out in a methanol-water solvent with the addition of 0.1% formic acid. During optimization of the HPLC protocol, the different stationary phases were probed: reversed phenyl-hexyl and C18, as well as a polar amide phase. The C18 phase proved to be the most suitable. In the chromatogram obtained at fluorimetric detection (Figure 2a), the signals with a retention time in the range of 13–17 min can be unambiguously attributed to CuVanc NCs since the other species are non-fluorescent at the excitation wavelength of 355 nm. As can be seen, a set of six dominant peaks is characteristic of CuVanc NCs, which relate to their different fractions. The revealed retention time indicates a pronounced hydrophobicity of CuVanc NCs, which is significantly higher in comparison with the initial reagents.

The chromatograms with diode-array detection were recorded at two wavelengths, 280 and 355 nm, which correspond to two absorption bands of CuVanc NCs. In the chromatogram registered at 280 nm (Figure 2b), the signals from the initial reagents and synthesis by-products appear in addition to the signals from the nanoclusters. Among the side signals, the most important peak is from vancomycin (4.5–6 min), which allows its reliable determination simultaneously with the nanoclusters. Two peaks at 8–10 min observed for the crude CuVanc NCs solution likely correspond to by-products of vancomycin oxidation during the nanocluster synthesis. It should be noted that copper ions, which also need to be monitored, elute at a dead time under the selected separation conditions (10% of phase B), which is typical for polar species in a reversed-phase mode. In the chromatogram registered at 355 nm (Figure 2c), only the peaks from the nanoclusters are observed.

Purification of metal nanoclusters from the excess reagents and byproducts is crucial for their further application in electrophoretic methods. Without this, it would be extremely difficult to distinguish the influence of nanoclusters and impurities during CE. The species requiring purification include excess unreacted copper sulfate and vancomycin together with vancomycin oxidation byproducts. Dialysis, ultrafiltration, ultracentrifugation, chromatography, precipitation, and combined methods are most commonly used to purify metal nanoclusters [58]. Surprisingly, solid-phase extraction is almost never used to purify metal nanoclusters, although it seems to be very suitable. Therefore, an SPE protocol for the purification of CuVanc NCs was developed at the next stage of the study. Based on the optimized HPLC conditions, the C18 phase was chosen as a sorbent, and mixtures of methanol and water in various ratios served as a mobile phase. At all stages, water, methanol or their mixture was used with the addition of 0.1% formic acid. The process consisted of the steps 1–5, which are represented in Figure 3 and described below, and took 40 to 50 min:

1. Conditioning for sorbent activation. At first, a cartridge was rinsed with 2 mL of methanol to remove contaminants and “straighten” C18H37 hydrocarbon radicals, which tend to bind via hydrophobic interactions in the absence of a non-polar phase. Next, 2 mL of water was passed through the cartridge to remove the excess organic solvent and bring the sorbent into equilibrium with the aqueous phase.

2. Loading. A total of 1 mL of the as-synthesized CuVanc NCs solution is slowly passed through the sorbent. The less polar CuVanc NCs were retained on the sorbent as a thin strip (2–3 mm wide), while a majority of the matrix passed through the sorbent and drained. It is believed that this step removes excess CuSO_4_.

3. Flushing with 2 mL of 20% methanol for elution of matrix components. This operation creates a gradient, similar to what happens in HPLC with a gradient elution. When the sorbent is treated with a 20% organic phase solution, vancomycin molecules unbound to the nanoclusters are eluted.

4. Eluting with 50% methanol for isolation of CuVanc NCs. The purified nanoclusters were eluted from the sorbent with 2 mL of a 50% methanol solution. Three fractions were collected: fraction I is a colorless solution collected before the nanoclusters elute; fraction II has a yellow-brown color and contains purified CuVanc NCs; and fraction III is a solution eluted after the nanoclusters (Figure 4a,b).

5. Washing with methanol to restore the sorbent. Finally, to clean the cartridge and prepare it for further use, 4 mL of methanol with the addition of 0.1% formic acid was passed through it.

**Figure 4:**
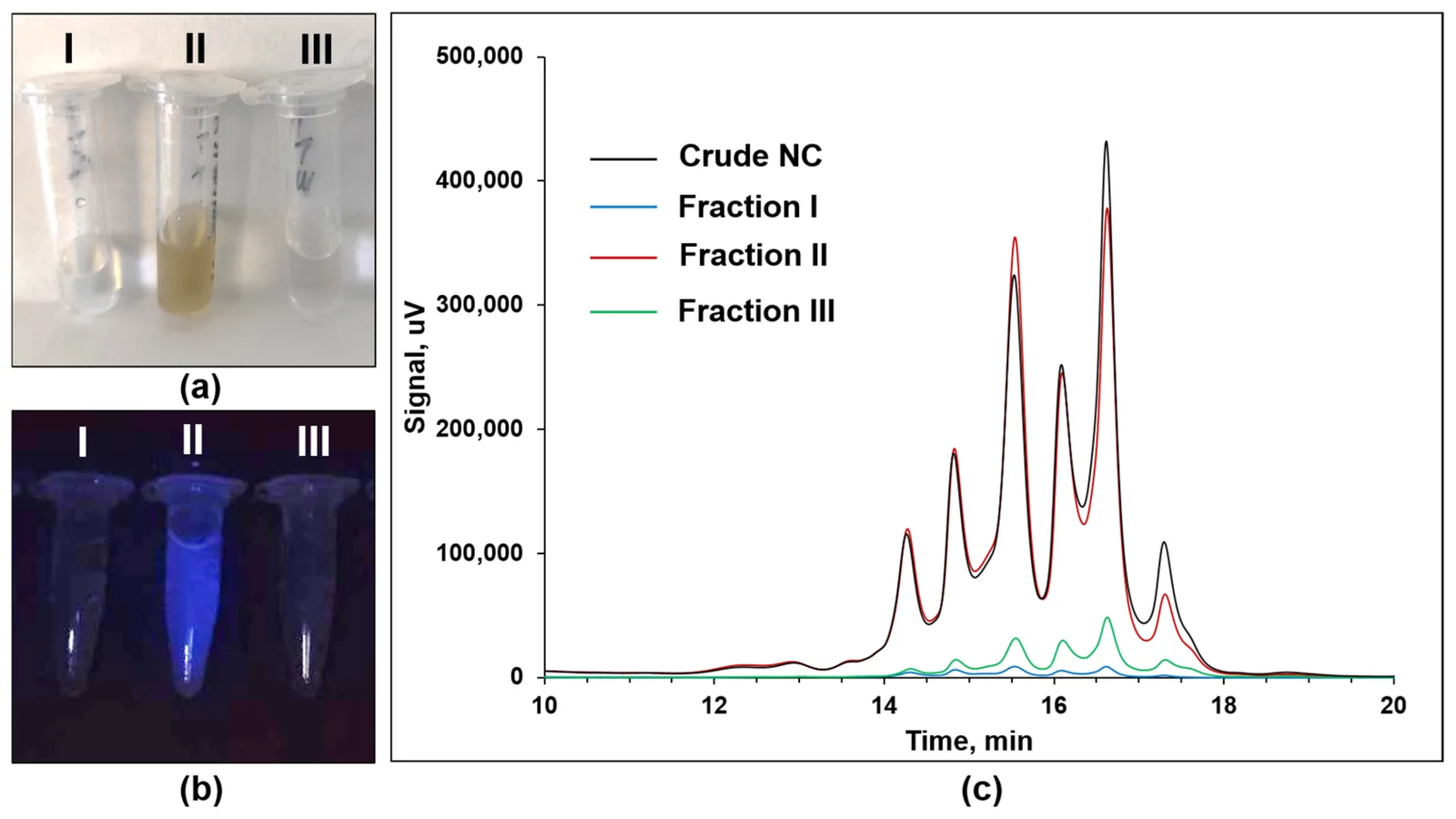
Photo of collected SPE fractions I–III under daylight (**a**) and ultraviolet (**b**); chromatograms of crude nanocluster solution and collected SPE fractions (**c**). Fluorescence detection: λ_ex_ = 355 nm, λ_em_ = 424 nm.

The preliminary quality control of SPE of CuVanc NCs was performed using HPLC-FlD. The chromatograms obtained at fluorescence parameters of 355/424 nm (excitation/emission wavelengths) demonstrate the preferential accumulation of the nanoclusters in fraction II (Figure 4).

At each SPE stage, a sample was taken to determine CuVanc NCs and vancomycin concentration by HPLC and to measure Cu^2+^ concentration by CE. Determination of vancomycin and CuVanc NCs concentration was performed in one analytical run. Because during method validation (see SI) it was confirmed that the total peak area of CuVanc NCs has a linear dependency on the dilution of the crude nanocluster solution, recovery (*R*) in each sample was calculated using the following formula:

(4) $$R\left(\mathrm{CuVanc}\right)=\frac{S}{S_{0}}\;\times \frac{V}{V_{0}}\times \frac{∆}{{∆}_{0}}\cdot 100\%,$$

where *S* is the total area of the nanoclusters signal in a sample, *S*_0_ is the total area of the nanoclusters signal in the crude solution, *V* is the sample volume, *V*_0_ is the volume of the crude nanoclusters solution loaded onto the cartridge, Δ is the sample dilution factor before analysis, and Δ_0_ is the dilution factor of the crude nanoclusters solution before analysis. The average nanocluster recovery was calculated based on five measurements and was found to be 90.9 ± 2.3% (*n* = 5, *p* = 0.95).

HPLC analysis of vancomycin was performed in a DAD regime at a detection wavelength of 280 nm. Calculation of vancomycin recovery in the samples obtained during SPE was performed according to the formula:

(5) $$R\left(\mathrm{Vanc}\right)=\frac{C}{C_{0}}\;\times \frac{V}{V_{0}}\times \frac{∆}{{∆}_{0}}\cdot 100\%,$$

where *C* is the concentration of vancomycin in a sample, *C*_0_ is the vancomycin concentration in the crude CuVanc NCs solution, *V* is the sample volume, *V*_0_ is the volume of the crude nanoclusters solution loaded onto the cartridge, Δ is the sample dilution factor before analysis, and Δ_0_ is the dilution factor of the crude nanoclusters solution before analysis. In the crude CuVanc NCs solution, only 0.73 ± 0.05 mM of free vancomycin was determined, which is about 2% of its initial concentration used in the synthesis. After SPE, the quantity of vancomycin in the CuVanc NCs solution was below 0.07 mM.

The samples were also analyzed by CE to determine the recovery of Cu^2+^ in different SPE fractions using Formula (5), similarly to vancomycin. In the crude CuVanc NCs solution, a concentration of Cu^2+^ was found to be 5.62 ± 0.14 mM, while the Cu^2+^ concentration was below LOD (0.003 mM) in fraction II (purified CuVanc NCs solution). The summarized results for the developed solid-phase extraction protocol are given in Table 1.

The collected fraction II with most of the CuVanc NCs was further concentrated 3–4 times by evaporation at 80 °C to remove residual methanol and formic acid, thereby minimizing solvent-related effects on the subsequent electrophoretic experiments. After the solvent removal, fluorescence was recorded and the measured intensity was used for normalization across the samples. The residual concentration of vancomycin in the concentrated CuVanc NCs solution after SPE was 0.13 ± 0.04 mM.

Although the proposed SPE method demonstrates high extraction efficiency, the potential impact of varying C18 column batches, flow rate fluctuations, and minor changes in solvent proportions on recovery was not experimentally evaluated. Therefore, a careful optimization of these parameters is recommended when adapting the method to different operational conditions or new cartridge batches.

### 3.2. Characterization of CuVanc Nanoclusters

Isolation of CuVanc NCs by SPE allowed for their comprehensive characterization. In particular, transmission electron microscopy was carried out, which is a direct method for visualization of metal nanoclusters. Figure 5 shows the HRTEM image obtained after the nanocluster purification. The copper nanoclusters are clearly visible; moreover, the edges of the crystal lattice are distinguishable at the highest magnification. The evaluated interplanar spacing of 0.21 nm is in agreement with a value characteristic of metallic copper. The average size of the synthesized CuVanc NCs is found to be 3.2 ± 0.6 nm. Thus, the need to purify metal nanoclusters must be emphasized once again, not only for their use but also for reliable characterization.

The size of CuVanc NCs was also analyzed using dynamic light scattering. The revealed value of 3.3 ± 1 nm is consistent with the TEM result. The zeta potential of CuVanc NCs determined in their water solution is +3.6 mV. This is provided by the ligand shell with a slightly positive net charge due to residual amino groups. Fourier-transform infrared and X-ray photoelectron spectroscopy were used to verify the composition of CuVanc NCs. The FTIR spectrum of the nanoclusters shows several signs of a slight change in the structure of vancomycin within their shell. In particular, these features include the intensity redistribution in the region of 3200–3600 cm^−1^ corresponding to N-H and O-H stretching vibrations and a decrease in the relative intensity of two bands at 1671 and 1129 cm^−1^ from the region of amide I and C-N stretching vibrations. These transformations may indicate that some of the amino groups within the vancomycin structure are affected during the formation of copper nanoclusters. In the XPS spectrum, two peaks centered at 932.8 and 952.6 eV can be attributed to Cu 2p3/2 and Cu 2p1/2 signals from Cu^0^, testifying to the nanocluster formation. The state of nitrogen was also established: the peak at 400.0 eV most likely corresponds to the amino group and the peak at 401.8 eV originates from a charged amino group. Thus, the absence of unreduced forms of copper, Cu(I) and Cu(II), in CuVanc NCs was confirmed.

### 3.3. Exploration of CuVanc Nanoclusters as a Chiral Selector in Capillary Electrophoresis

Purified CuVanc NCs were evaluated as a chiral selector in capillary electrophoresis, which was started with the examination of how CuVanc NCs affect the electroosmotic flow (EOF) direction and mobility across different pH values at a fixed concentration of the nanoclusters (Figure 6a). This first stage is methodologically crucial because EOF is a primary determinant of migration behavior, resolution, and analysis time in CE. An EOF from cathode to anode is observed at pH 2, similar to vancomycin alone, which is likely provided by protonated amino groups. Under alkaline conditions, EOF changes direction from anode to cathode (becomes cathodic) and its mobility decreases compared with both the background electrolyte without additives and the background electrolyte with vancomycin alone. Consequently, CuVanc NCs appear to adsorb more effectively onto the capillary surface and shield silanol groups more efficiently. Summarizing the described EOF behavior, it can be hypothesized that CuVanc NCs form a dynamic coating on the inner wall of the fused-silica capillary. To verify this assumption, a direct study of the inner capillary surface is required.

The dependence of EOF mobility on the nanocluster concentration was also examined (Figure 6b). The saturation of the capillary surface was reached for a 10-time diluted solution of CuVanc NCs relative to the solution after SPE. This indicates that even a relatively low concentration of CuVanc NCs provides a sufficient coverage to occupy the accessible adsorption sites on the fused-silica wall. A further increase in the nanocluster concentration gave a little additional change in EOF. This behavior is consistent with interfacial electrokinetic theory, according to which EOF is controlled primarily by the effective surface charge and structure of the electrical double layer rather than by bulk additive concentration once the surface layer is established.

Since vancomycin has been widely established in CE as an effective chiral selector for nonsteroidal anti-inflammatory drugs (NSAID) enantiomers [54], CuVanc NCs were explored using this class of compounds. The test mixture was further expanded by addition of acenocoumarol and amlodipine, allowing assessment of the selector toward pharmaceuticals with different structural and ionization properties. This choice is methodologically justified because the vancomycin-based chiral recognition is known to rely on multiple interaction modes and has previously shown strong performance for acidic racemates [54,55,56], particularly arylpropionic NSAIDs.

To provide the experimental integrity, a chiral recognition of the selected analytes was firstly investigated in CE using vancomycin by itself. The analysis was performed in a 50 mM phosphate background electrolyte at pH values of 6, 7, and 8 and at vancomycin concentrations of 0.05, 0.1, 0.25, and 0.5 mM. This range was chosen based on the residual vancomycin concentrations found in the solution of CuVanc NCs after SPE. Among the conditions used, the highest value of enantioselectivity was achieved at the lower pH of the background electrolyte and the highest concentration of vancomycin. However, when the vancomycin concentration was only 0.1 mM, the baseline separation of ketoprofen and partial separation of flurbiprofen were observed (Figure 7). In the case of acenocoumarol, the chiral separation was noted at a vancomycin concentration greater than 0.25 mM. Notably, the enantioseparation was detected only for the acidic analytes, whereas amlodipine was not resolved under the conditions employed. This result agrees with the well-established preference of vancomycin-based CE systems for anionic species, for which electrostatic contributions play a central role in chiral discrimination.

The preliminary evaluation of chiral separation with vancomycin allowed us to move on to the CE experiments with CuVanc NCs. When the nanoclusters were added to the background electrolyte even at low concentration, the chiral separation pattern changed remarkably. Under these conditions, the enantiomers of ketoprofen and flurbiprofen remained almost unseparated, whereas amlodipine became enantioresolved (50 mM PBS pH 6.0, CuVanc NCs 5% (*v*/*v*) relative to the solution after SPE, *α* = 1.017, *R_s_* = 0.91, *N* = 340–400 th. t.p.) (Figure 8). However, a further increase in the pH of the background electrolyte led to a noticeable decrease in the enantiomeric separation selectivity of amlodipine; consequently, at pH 8.0, the enantiomeric peaks completely merged, preventing chiral resolution. Furthermore, an increase in the concentration of CuVanc NCs resulted in a decline in separation efficiency. At lower pH values (pH 6.0–7.0), amlodipine is extensively protonated, favoring strong electrostatic binding with the selector. Raising the pH to 8.0 reduces its degree of protonation, which substantially weakens the analyte–selector interaction; consequently, enantioselectivity decreases. Importantly, because the electroosmotic flow exhibits only minor variation across this pH range, the suppression of enantioseparation is predominantly governed by the altered protonation state of the analyte rather than changes in EOF velocity.

In the case of acenocoumarol, a distinct trend was observed. At pH 6.0, the analyte was not detected under either polarity, even with an extended analysis time of 60 min. Under these conditions, its effective electrophoretic mobility is highly likely equal in magnitude but opposite in direction to the electroosmotic flow (EOF), resulting in a near-zero net migration velocity along the capillary. Increasing the background electrolyte pH to 7.0 significantly enhanced the negative charge and effective mobility of acenocoumarol, thereby enabling its reliable detection under negative polarity (Figure 9). Furthermore, systematically increasing the CuVanc NC concentration from 10% to 20% (*v*/*v*) and raising the buffer pH from 7.0 to 8.0 led to a progressive increase in enantioselectivity and an improvement in peak resolution (Table 2). However, raising the pH to 8.0 was accompanied by a noticeable decrease in separation efficiency. Consequently, the optimal balance between high efficiency and near-baseline resolution was achieved using 50 mM PBS pH 7, CuVanc NC 20% (*α* = 1.015, *R_s_* = 0.87, *N* = 140–170 th. t.p.).

In our view, the chiral separation of acenocoumarol is predominantly driven by non-electrostatic interactions rather than ionic forces. As the background electrolyte pH is increased to 7.0–8.0, both the acenocoumarol enantiomers and the CuVanc NCs become negatively charged, giving rise to mutual electrostatic repulsion. Under these conditions, the stereoselective binding is primarily mediated by other types of forces, such as hydrophobic interactions, π-π stacking, and hydrogen bonding. Furthermore, it is plausible that the suppression of strong electrostatic interactions at elevated pH actually benefits enantioseparation: by minimizing nonspecific ionic forces, the contribution of secondary, highly stereoselective interactions becomes predominant. The spatial steric constraints within the nanocluster’s chiral shell ensure differential binding affinities for the two enantiomers despite the overall electrostatic barrier. Thus, in the case of acenocoumarol, the BGE pH primarily serves to ensure sufficient negative ionization and effective electrophoretic mobility for reliable migration and detection, while enantioselectivity itself is dictated by non-electrostatic interactions.

In contrast, for ketoprofen and flurbiprofen, neither varying the BGE pH nor increasing the concentration of the nanoclusters in the background electrolyte enabled enantiomeric separation. The weak trace resolution observed for analytes at pH 6.0 can be partially attributed to residual traces of unreacted or desorbed free vancomycin present in the purified nanocluster solution. Although SPE effectively removes the bulk synthetic matrix, trace levels of molecular vancomycin may persist and contribute independently to initial chiral recognition.

The revealed significant differences in the effects of vancomycin and its copper nanoclusters on the chiral separation of test compounds indicate that metal atoms likely alter an active chiral-recognition region of vancomycin. Native vancomycin exhibits a high enantioselectivity because it provides a dense array of hydrogen-bonding, aromatic, hydrophobic, and amide-containing interaction sites. Because CuVanc NCs were synthesized without an external reducing agent, it is plausible that functional groups of vancomycin involved in the chiral recognition are chemically modified or become less accessible in the nanoclusters. This can explain why native vancomycin’s strong discrimination toward acidic analytes such as ketoprofen and flurbiprofen is markedly reduced, while a new binding environment becomes available that is capable of recognizing amlodipine enantiomers, which were not resolved in the presence of vancomycin alone.

A second mechanistic consequence is related to the nanoclusters’ adsorption on the inner capillary wall. As a consequence, CuVanc NCs are in two states inside the capillary: as a coating adsorbed on the wall and as dispersed species in the background electrolyte (pseudostationary phase). Therefore, analytes can distribute between free solution and NCs adsorbed on the capillary wall. Under these conditions, the system no longer works solely in the electrophoretic regime, but rather in the electrochromatography regime. Consequently, the separation depends not only on electrophoretic mobility, but also on differential partitioning between these two interaction environments. Moreover, the formation of this adsorbed coating substantially alters the surface charge (zeta potential), thereby modulating the electroosmotic flow, which further contributes to the overall separation mechanism. The distinct enantioseparation behaviors observed for amlodipine and acenocoumarol, contrasted with the lack of resolution for ketoprofen and flurbiprofen, clearly substantiate the fundamental role of the nanoclusters as true chiral selectors.

## 4. Conclusions

Vancomycin-stabilized copper nanoclusters with a size of ~3 nm were successfully synthesized through a bottom-up technique using lyophilized antibiotic and copper sulfate as initial reagents. The nanocluster formation was confirmed by fluorescence spectroscopy, transmission electron microscopy, dynamic light scattering, and X-ray photoelectron spectroscopy. For further use in capillary electrophoresis, as-synthesized CuVanc NCs were purified and isolated by solid-phase extraction. The designed SPE protocol gave a recovery of the nanoclusters over 90% at a treatment time of 40 min only. Importantly, SPE allowed obtaining the purified CuVanc NCs solution in a quantity sufficient for numerous electrophoretic experiments.

The examination of the nanoclusters in capillary electrophoresis was performed using a mixture of model drugs of different nature. Chiral separation was observed for both positively and negatively charged analytes and the best selectivity coefficients were obtained for amlodipine and acenocumarol. The comparative CE experiments with vancomycin made it possible to unambiguously identify the chiral selector effect of the nanoclusters. The EOF behavior observed at different concentrations of CuVanc NCs presumes the formation of a dynamic coating on the inner wall of the fused-silica capillary. The assumed dynamic coating apparently contributes to the achieved chiral separation. The demonstrated ability of CuVanc NCs to act as an effective chiral selector in capillary electrophoresis is a fundamental step which may contribute to the diversifying applications of metal nanoclusters.

Notwithstanding the promising analytical potential of the proposed CuVanc NC-based enantioseparation system, several limitations of the present study should be acknowledged:

- Although purified CuVanc NC preparations were employed, liquid chromatographic separation revealed the presence of several distinct sub-structures (six primary signal peaks). Consequently, it remains unresolved which specific nanocluster species are principally responsible for the observed chiral recognition.
- Identification of all signals observed in the chromatogram currently remains an unresolved challenge; however, active research and methodical efforts are presently underway to fully characterize these individual species.
- The SPE purification protocol was optimized on a specific C18 sorbent chemistry. The effects of batch-to-batch variability, sorbent manufacturers, and minor fluctuations in solvent proportions across wash and elution steps were not systematically evaluated. While minor adjustments may be necessary when transferring the protocol across different sorbent suppliers, these variations are expected to be incremental rather than fundamental.
- The chiral recognition capabilities of CuVanc NCs were demonstrated on a selected group of pharmaceutical drugs. Testing on real biological or complex matrix samples was beyond the scope of this work, as bioanalytical application and method validation represent a distinct, dedicated stage of analytical development.
- A complete, detailed molecular mechanism of enantioseparation was not fully established. Fundamental mechanistic studies require significantly larger volumes and higher concentrations of isolated, monodisperse NC fractions, along with advanced spectroscopic and computational methods. Furthermore, explicit knowledge of the exact thermodynamic mechanism is not strictly mandatory for addressing practical routine challenges in applied chiral separations.

These shortcomings demonstrate a need for further research of copper nanoclusters in chiral CE, which would bring new insightful details and analytical performance, hopefully.

In summary, the performed proof-of-concept study can serve as a starting point for further research paths addressed to the implementation of metal nanoclusters in chiral separation: (i) assessment of copper nanoclusters stabilized with other organic ligands and nanoclusters with gold or silver cores; (ii) developing capillary electrochromatography stationary phases; and (iii) testing real pharmaceutical, environmental or biological samples. The benefits of ligand shell variability and high density of ligands on a metal core make the nanoclusters worthy competitors to traditional molecular chiral selectors.

## Figures and Tables

**Figure 1:**
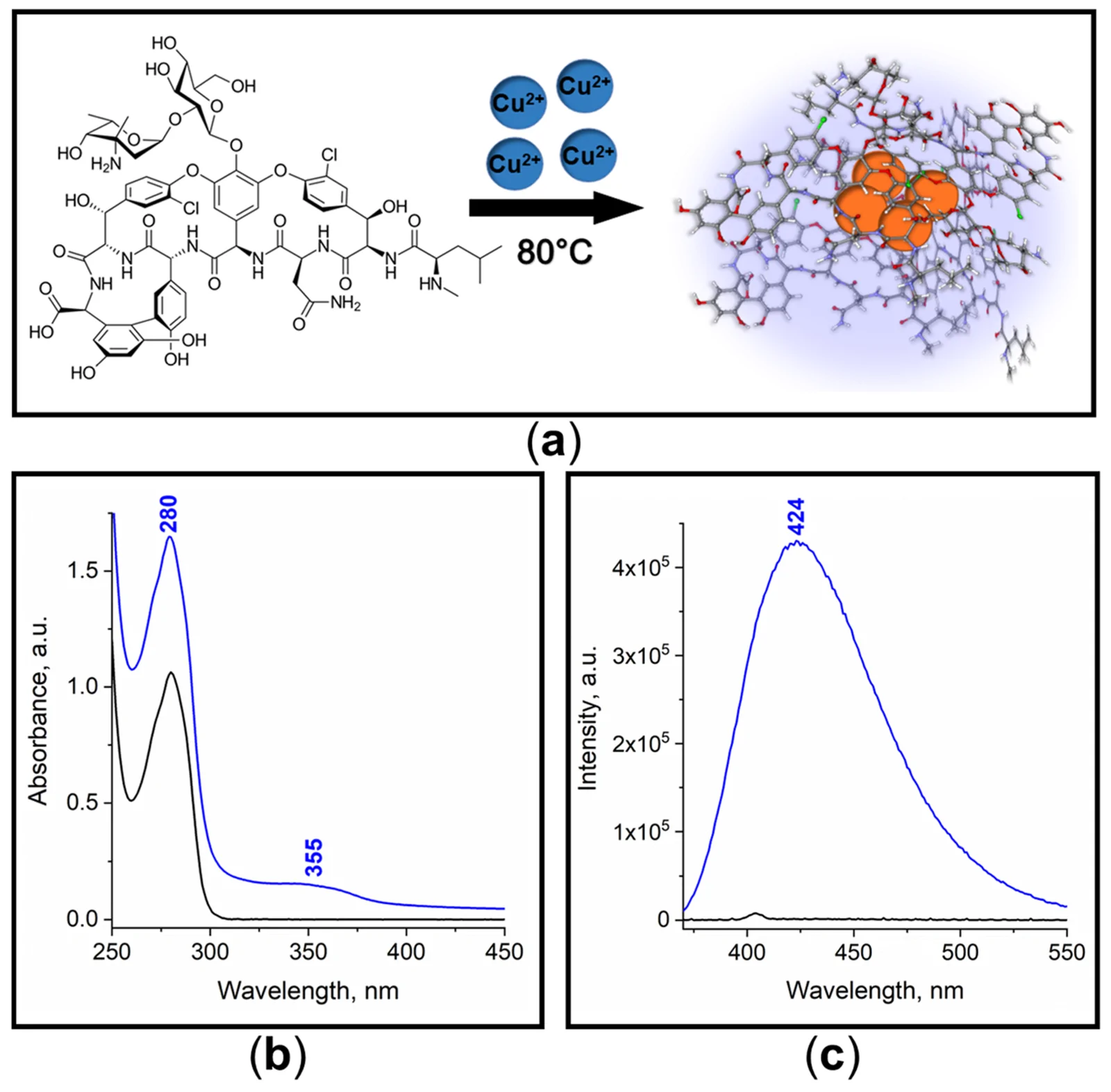
Schematic representation of formation (**a**) and absorption and emission spectra of fluorescence (**b**,**c**) of vancomycin-stabilized copper nanoclusters (blue), λ_ex_ = 355 nm. The spectra of vancomycin are shown for comparison (black).

**Figure 2:**
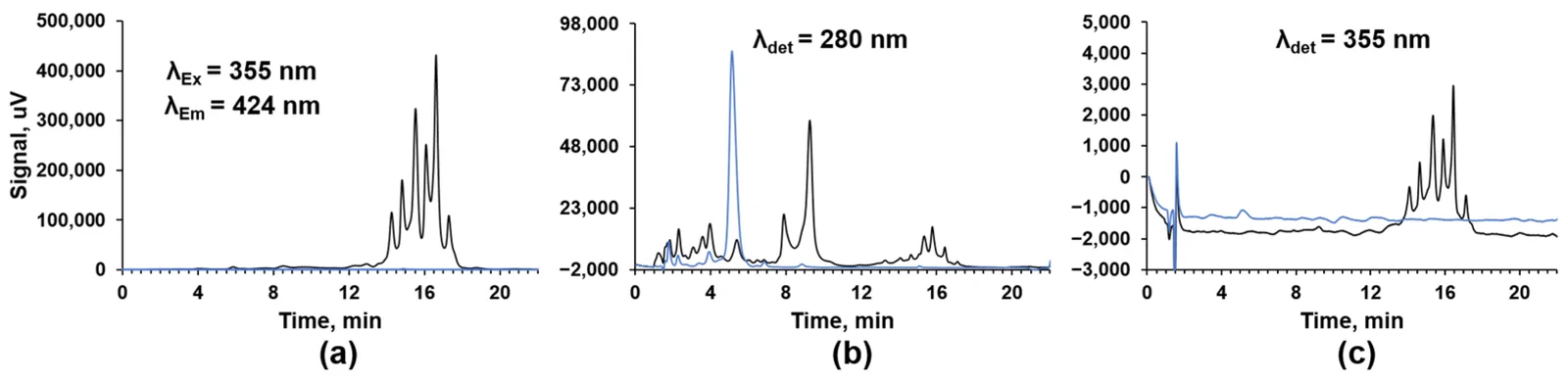
Chromatograms of 0.5 mM vancomycin (black) and crude CuVanc NCs solution (blue) at different regimes of detection: fluorescence (**a**) and diode-array detection (**b**,**c**).

**Figure 3:**
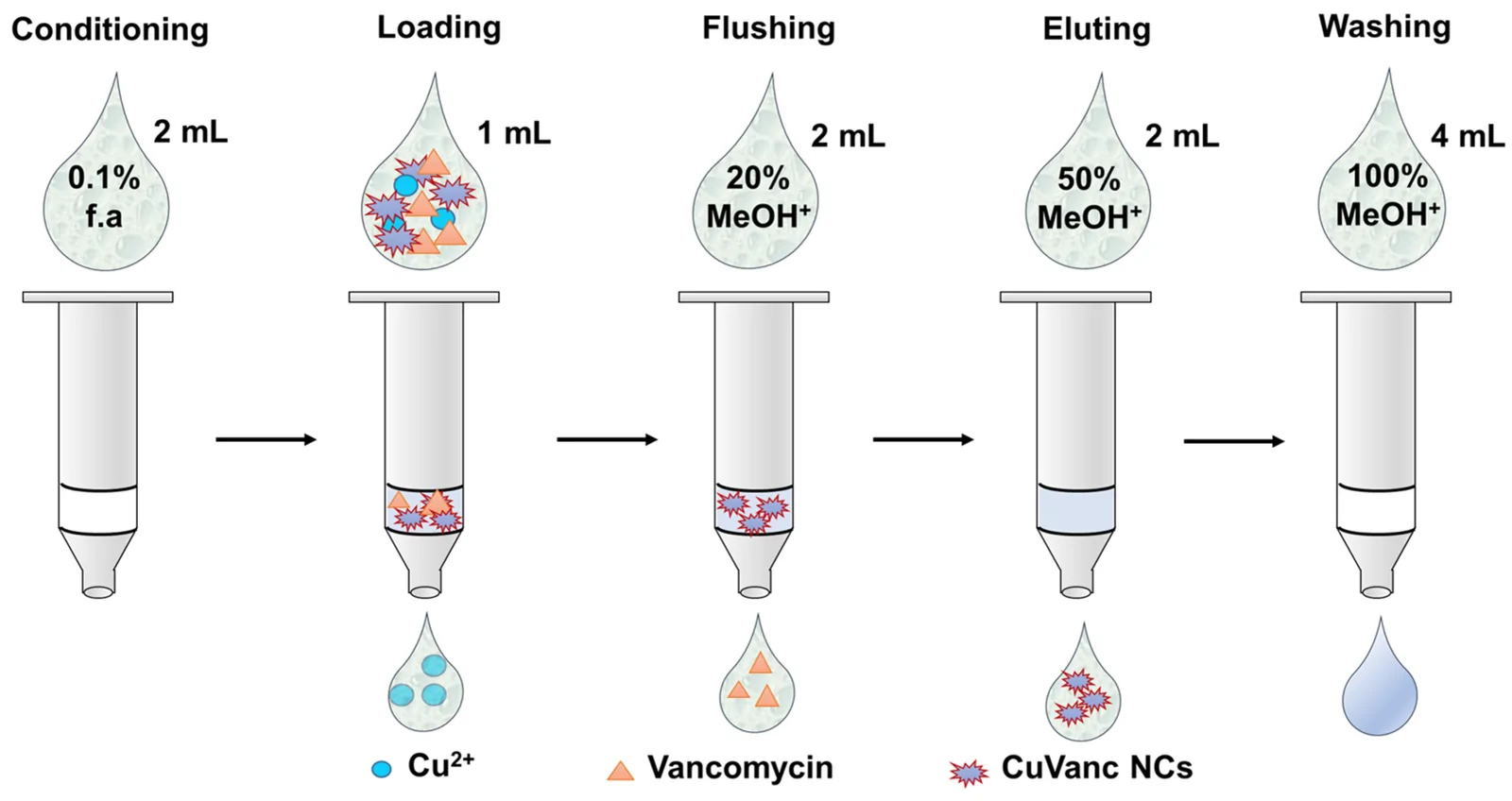
Scheme of CuVanc NCs solid-phase extraction. A plus sign near methanol indicates its acidification by formic acid (0.1% *v*/*v*).

**Figure 5:**
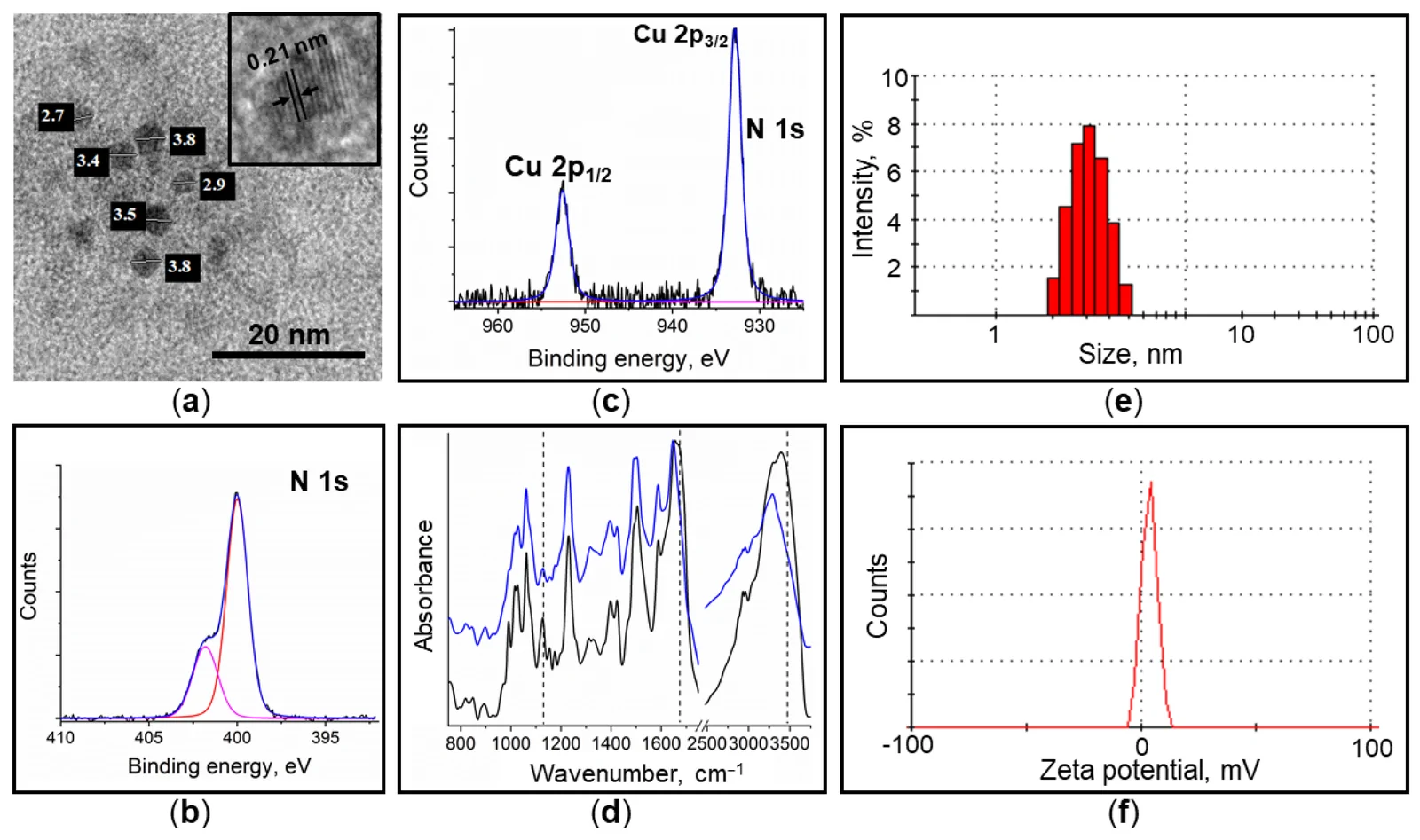
(**a**) HRTEM image, (**b**,**c**) XPS, (**d**) FTIR spectrum (blue), (**e**) hydrodynamic size, and (**f**) zeta potential of purified CuVanc NCs. FTIR spectrum of vancomycin in (**d**) is given for comparison.

**Figure 6:**
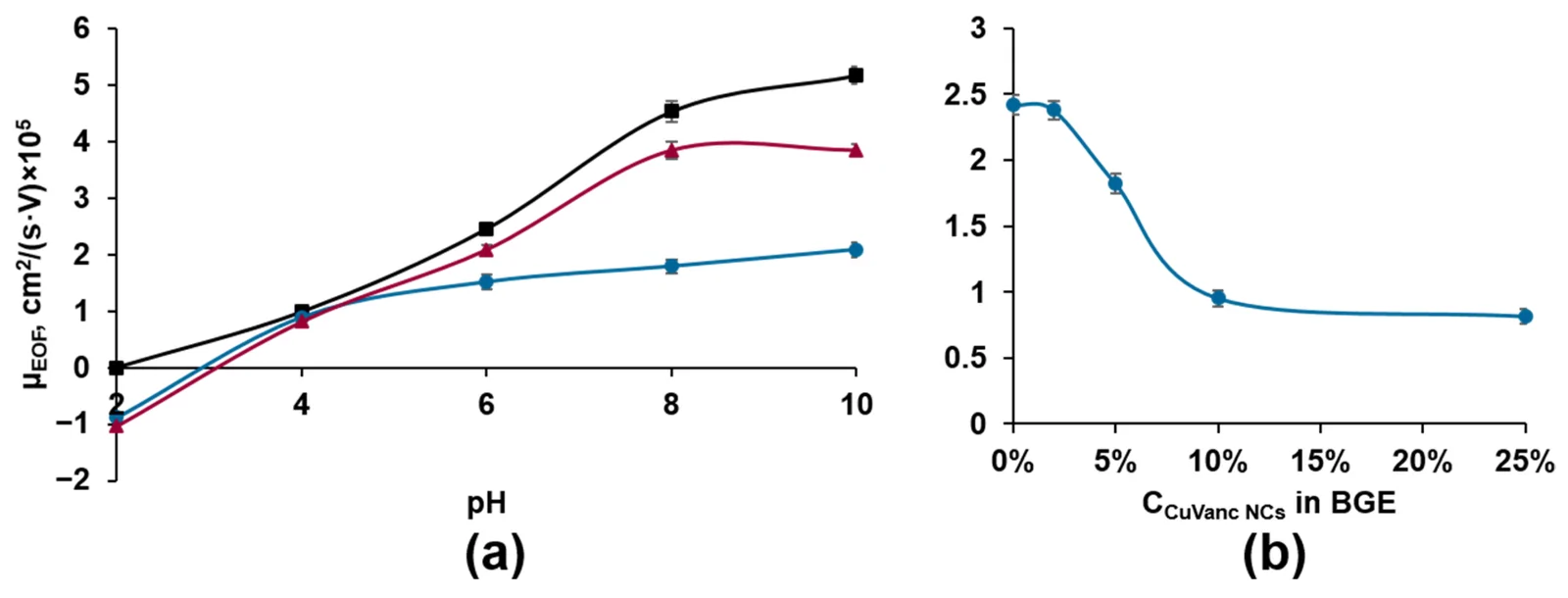
(**a**) Dependence of EOF on pH in phosphate-based background electrolyte of different compositions: without additives (black), with 1 mM vancomycin (red), and with CuVanc NCs (blue); (**b**) Dependence of EOF on volume fraction of CuVanc NCs in 50 mM PBS at pH 6.0. The volume fraction of CuVanc in BGE is given relative to the dilution of the NC solution after SPE (25% (*v*/*v*) corresponds to a 4-fold dilution, etc.). The EOF marker is 0.05% DMF.

**Figure 7:**
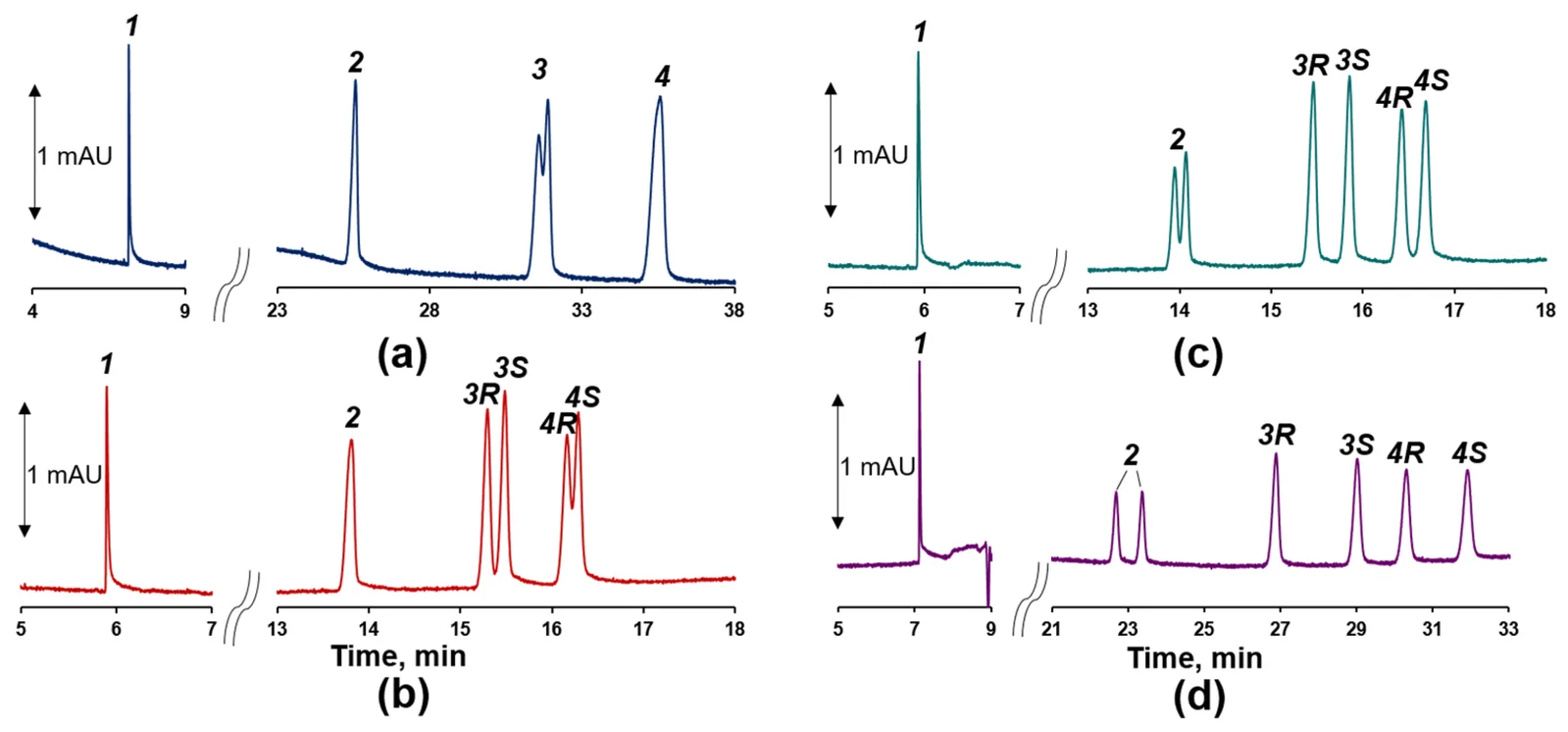
Electropherogram of working solution consisting of amlodipine (1), acenocoumarol (2), ketoprofen (3), flurbiprofen (4), and different concentrations of vancomycin: (**a**) 0.05, (**b**) 0.1, (**c**) 0.25, (**d**) 0.5 mM. BGE: 50 mM PBS, pH 6.0, U = +20 kV.

**Figure 8:**
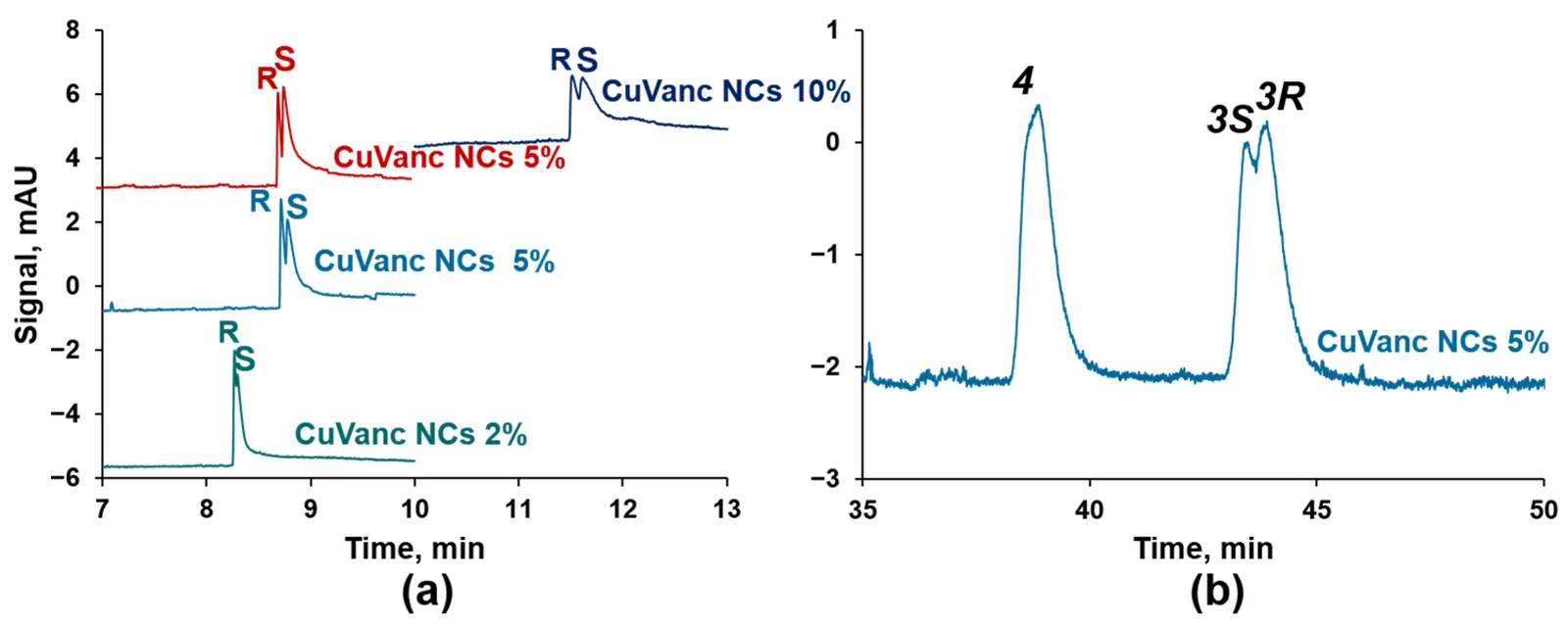
Electropherograms of working solution: (**a**) amlodipine at different volume fractions of CuVanc NCs in 50 mM PBS pH 6.0, U = +20 kV, red line with addition of pure S-amlodipine for enantiomer identification; (**b**) flurbiprofen (4) and ketoprofen (3) in 50 mM PBS pH 6.0, CuVanc NCs, 5% (*v*/*v*), U = −20 kV.

**Figure 9:**
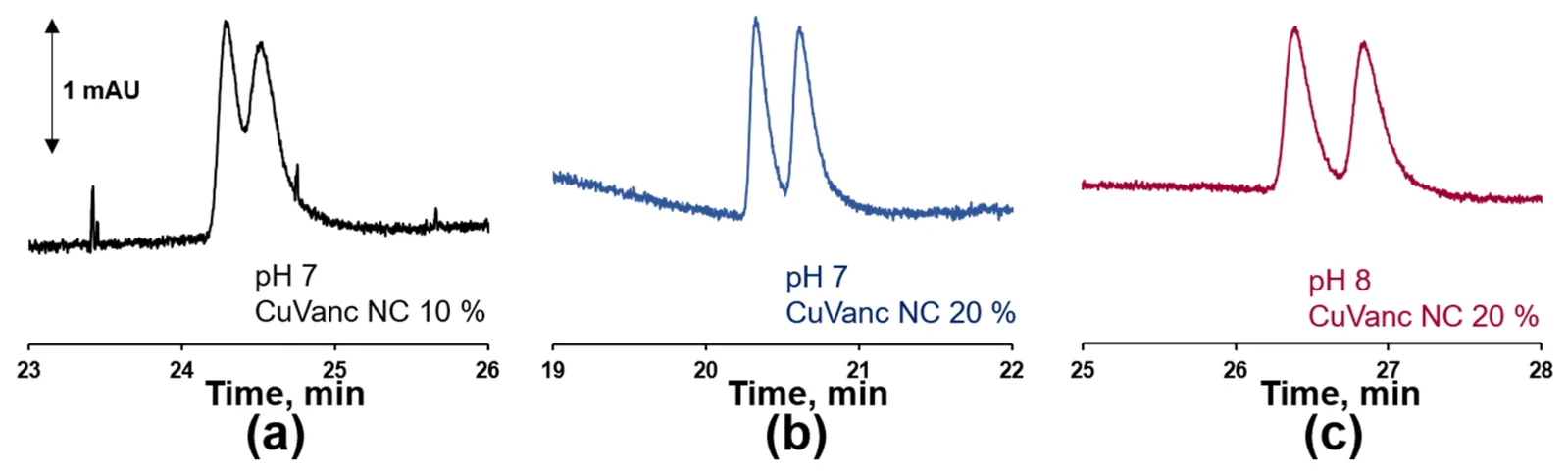
Electropherograms of acenocoumarol working solution in different BGE: (**a**) 50 mM PBS pH 7 + CuVanc NCs 10% (*v*/*v*), (**b**) 50 mM PBS pH 7 + CuVanc NCs 20% (*v*/*v*), (**c**) 50 mM PBS pH 8 + CuVanc NCs 20% (*v*/*v*); U = −20 kV. Detection wavelength was 315 nm.

**Table 1:** Recovery *R* (%) of CuVanc NCs, vancomycin and Cu^2+^ at different stages of solid-phase extraction (*n* = 5, *p* = 0.95).

SPE Stage		*R* (CuVanc), %	*R* (Vanc), %	*R* (Cu^2+^), %
Conditioning		0	0	0
Loading		0	0.1 ± 0.1%	60.3 ± 3.2
Flushing		0.3 ± 0.2	75.9 ± 4.0%	37.1 ± 2.5
Eluting	fraction I	3.8 ± 3.8	20.2 ± 6.6%	1.2 ± 0.1
	fraction II	91.2 ± 2.2	6.9 ± 2.4%	0
	fraction III	8.6 ± 4.2	1.3 ± 1.3%	0
Washing		1.7 ± 2.1	0	0

**Table 2:** Electrophoretic parameters of acenocoumarol enantiomer separation under different conditions.

Parameters	50 mM PBS pH 7, CuVanc NC 10%	50 mM PBS pH 7, CuVanc NC 20%	50 mM PBS pH 8, CuVanc NC 20%
Enantioselectivity (*α*)	1.010	1.015	1.017
Efficiency (*N*), th. t.p.	140–190	140–170	110–130
Resolution (*R_s_*)	0.76	0.87	0.90

## Data Availability

Data will be made available on request.

## Supplementary Materials

The following supporting information can be downloaded at https://www.mdpi.com/article/10.3390/nano16211361/s1. Figure S1: (a) calibration curve for vancomycin at concentration range 0.005–1.00 mM and (b) chromatograms of solution with 0.005 mM (LLOQ) of vancomycin (black line) and water samples (color line) for assessment of selectivity; Table S1: accuracy and precision of vancomycin determination; Table S2: preparation of calibration solutions for CuVanc NC; Figure S2: chromatograms of calibration solution of crude NC (a) and calibration curve (b); Figure S3: calibration curve (a) with calibration (black squares) and quality control (blue triangles) samples and electropherograms (b) of LLOQ samples (black line) and collected fraction after flushing of the cartridge with 20% MeOH acidified by 0.1% formic acid (red line); Table S3: accuracy and precision of the method for Cu^2+^ determination.

## Abbreviations

The following abbreviations are used in this manuscript:

NC	Nanocluster
CuVanc NCs	Vancomycin-stabilized copper nanoclusters
CE	Capillary electrophoresis
HPLC-FlD-DAD	High-Performance Liquid Chromatography coupled with Fluorescence Detection (FLD) and Diode Array Detection
SPE	Solid-phase extraction
HRTEM	High-resolution transmission electron microscopy
DLS	Dynamic light scattering
XPS	X-ray photoelectron spectra
NSAID	Nonsteroidal anti-inflammatory drugs
EOF	Electroosmotic flow
PBS	Phosphate-buffered solution
BGE	Background electrolyte
