# Supplementary material for: Vancomycin-Stabilized Copper Nanoclusters: Isolation by Solid-Phase Extraction and Application in Chiral Capillary Electrophoresis
Source: Nanomaterials (Basel). 2026 Oct 26;16(21):1361. doi: 10.3390/nano16211361 (PMC13765248; doi:10.3390/nano16211361)
Supplement: Supplementary file 1 [file nanomaterials-16-01361-s001.zip › nanomaterials-4648358-supplementary.pdf]

Supplementary Materials

Vancomycin-stabilized copper nanoclusters: isolation by sol-id-phase extraction and application in chiral capillary electro-phoresis

E. Kolobova, A. Demenshin, Y. Zimarina, E. Solovyeva

Institute of Chemistry, Saint-Petersburg State University, Universitetskaya nab. 7/9, 199034 Saint-Petersburg, Russia

The methods for determination of copper, vancomycin, and nanocluster were partially validated to ensure reliability of the results while monitoring each step of the sample preparation process. The following parameters were assessed during the partial validation: linearity, limit of quantification, detection limit, selectivity, accuracy, and intraday and interday precision.

At method validation, the quality controls (QC) samples for accuracy and precision runs were prepared at a minimum of 4 concentration levels within the calibration curve range: the lower limit of quantitation (LLOQ), within three times of the LLOQ (QCA), 50% of the calibration curve range (QCB) and at 75% of the upper limit of quantification (QCC).

Accuracy and precision of the method was evaluated by injecting the series of quality control samples at 4 concentration levels. Method accuracy was determined as an average percentage, calculated by dividing the measured concentration by the nominal concentration for each QC sample. Precision was evaluated as the percentage relative standard deviation (% RSD) across replicate injections.

Following standard regulatory guidelines [61,62], the acceptance criteria required accuracy to remain within ±15% of the nominal concentration, with the exception of the LLOQ, where ±20% deviation was permitted. Similarly, precision (%RSD) at each concentration level was not to exceed 15%, with the allowance of up to 20% at the LLOQ.

Validation of a HPLC-DAD method for determination of vancomycin

Stock solutions of vancomycin was prepared in water to achieve a concentration of 20 mM of vancomycin. Calibration solutions were prepared by diluting the corresponding stock solutions in water to obtaine a final concentration of 0.005, 0.010, 0.025, 0.050, 0.100, 0.250, 0.500 and 1.000 mM. Quality control samples were prepared by diluting the corresponding stock solutions in water to achieve a final concentration of 0.005 (LLOQ), 0.015 (QCA), 0.500 (QCB), 0.750 (QCC) mM.

The separation was carried out in the gradient elution mode: starting from 10% phase B, increasing the concentration of phase B from 10 to 15% over 0–3 min holding for 5 min at 15% phase B, then increasing the concentration of phase B from 15 to 35% over 8–13 min, holding from 13 to 16 min at 35% phase B, then increasing the concentration of phase B from 35 to 100% over 16–19 min, holding from 19 to 22 min at 100% phase B and returning to the initial conditions in 0.1 min, followed by conditioning the system for 5 min. Sample injection was 5 uL.

Calibration was carried out using 8 points. Evaluated methods are linear between 0.005 – 1.000 mM of vancomycin. The calibration curves were represented by the following linear regression equations: y = 4987658x-6914 (R^2=0.9997) (Fig. S1a): Weight:1/x. Lower limit of quantification (LLOQ) of the method was 0.005 mM (Fig 1b). The limit of quantitation (LOQ) was determined based on a signal-to-noise ratio. The LOQ was defined as the lowest concentration of vancomycin that produced a peak with an S/N ratio of 10:1. LOQ was 0.003 mM. The limit of detection was defined as the lowest concentration of the analyte that produced as S/N ratio of 3:1. The LOD was verified as 0.001 mM.

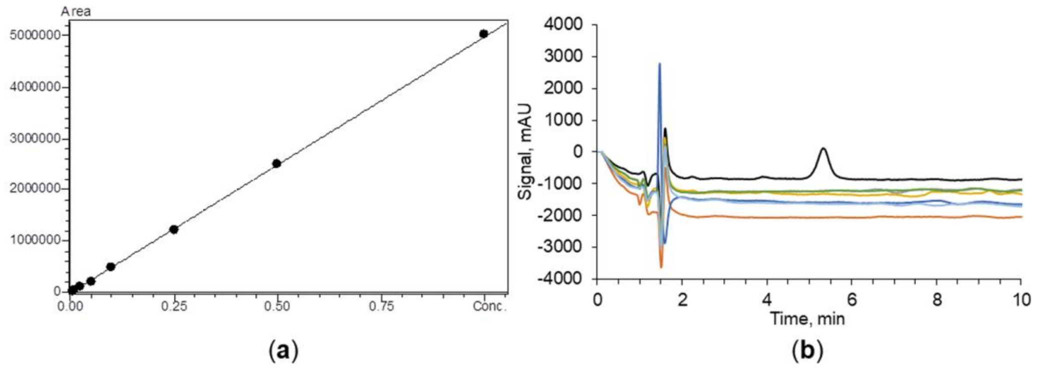

Figure S1. (**a**) Calibration curve for vancomycin at concentration range 0.005 – 1.00 mM and (**b**) chromatograms of solution with 0.005 mM (LLOQ) of vancomycin (black line) and water samples (color line) for assessment of selectivity.

Selectivity was determined by analyzing a minimum of six water samples. Chromatograms showed no interfering peaks at the vancomycin's retention time (Fig. S1b).

Efficiency for vancomycin was 8600±400 t.p./m.

The presicion of vancomycine determination within a single run ranged from 0.1% to 1.9% and between runs from 0.8% to 2.8%, with an accuracy of 92.5% to 118.8% and 96.0% to 113.1%, respectively (Table S1).

Table S1. Accuracy and precision of vancomycin determination.

		LLOQ (0.005 mM)					Average	RSD, %
Run 1	C, mM	0.0056	0.0055	0.0055	0.0055	0.0056	0.0055	0.9%
	Accuracy, %	112.2%	110.0%	110.6%	109.8%	111.0%	110.7%	
Run 2	C, mM	0.0058	0.0060	0.0058	0.0058	0.0058	0.0058	1.5%
	Accuracy, %	116.2%	120.0%	116.2%	116.0%	115.6%	116.8%	
Run 3	C, mM	0.0055	0.0055	0.0057	0.0057	0.0055	0.0056	1.9%
	Accuracy, %	110.6%	110.0%	114.8%	113.0%	110.2%	111.7%	
Inter-day Accuracy = 113.1%, RSD = 2.8%								

		QCA (0.015 mM)					Average	RSD, %
Run 1	C, mM	0.0146	0.0148	0.0147	0.0149	0.0148	0.0148	0.8%
	Accuracy, %	97.4%	98.6%	97.9%	99.3%	98.7%	98.4%	
Run 2	C, mM	0.0145	0.0146	0.0146	0.0145	0.0146	0.0146	0.4%
	Accuracy, %	96.9%	97.5%	97.6%	96.7%	97.3%	97.2%	
Run 3	C, mM	0.0140	0.0139	0.0139	0.0138	0.0138	0.0139	0.6%
	Accuracy, %	93.3%	92.3%	92.9%	92.0%	92.1%	92.5%	
Inter-day Accuracy = 96.0%, RSD = 2.8%								

		QCB (0.500 mM)					Average	RSD, %
Run 1	C, mM	0.503	0.501	0.503	0.504	0.496	0.501	0.6%
	Accuracy, %	100.6%	100.1%	100.6%	100.7%	99.2%	100.2%	
Run 2	C, mM	0.502	0.503	0.500	0.505	0.497	0.502	0.6%
	Accuracy, %	100.4%	100.6%	100.0%	101.1%	99.5%	100.3%	
Run 3	C, mM	0.494	0.491	0.495	0.487	0.487	0.491	0.7%
	Accuracy, %	98.8%	98.2%	99.0%	97.5%	97.4%	98.2%	
Inter-day Accuracy = 99.6%, RSD = 1.2%								

		QCC (0.750 mM)					Average	RSD, %
Run 1	C, mM	0.755	0.755	0.757	0.757	0.757	0.756	0.1%
	Accuracy, %	100.7%	100.7%	100.9%	100.9%	100.9%	100.8%	
Run 2	C, mM	0.750	0.766	0.751	0.768	0.760	0.759	1.1%
	Accuracy, %	100.0%	102.2%	100.2%	102.4%	101.4%	101.2%	
Run 3	C, mM	0.743	0.756	0.755	0.759	0.748	0.752	0.8%
	Accuracy, %	99.1%	100.8%	100.6%	101.1%	99.7%	100.3%	
Inter-day Accuracy = 100.8%, RSD = 0.8%								

Validation of a HPLC-FlD method for determination of relative concentration of CuVanc NCs

Classical validation is complicated first by the lack of certified nanocluster standards, which limits direct assessment of trueness and traceability, and second by the multicomponent nature of nanocluster samples, in which discrete subpopulations can differ measurably in size, composition, fluorescence, and chromatographic behavior.

Since nanocluster determination was required to calculate recovery, defined as the ratio of nanocluster concentration measured in the sample to the initial nanocluster content in the crude preparation, it was essential to verify that the fluorescence response was proportional to the nominal nanocluster concentration within the working range. For this purpose, the crude nanocluster dispersion was diluted stepwise, and calibration plots were constructed for each fluorescence-detected signal eluting between 11 and 19 min. This step was particularly important because crude nanocluster samples are typically heterogeneous mixtures composed of several emissive fractions with discrete chromatographic and optical properties rather than a single uniform species. Demonstrating linearity for signal therefore confirmed that fluorescence could serve as a valid relative quantitative measure for nanocluster fraction and provided the analytical basis for subsequent recovery calculation.

Calibration solutions (CS) of NCs were prepared by diluting crude CuVanc NCs solution according to Table S2.

Table S2. Preparation of calibration solutions for CuVanc NC.

	CS1	CS2	CS3	CS4	CS5	CS6
$V_{NCs\ crude}$, uL	10	20	40	80	120	160
H_2O_{DI}, uL	990	980	960	920	880	840
Nominal concentration	0.01	0.02	0.04	0.08	0.12	0.16

Calibration was carried out using 6 points. For each point, 3 measurements were made. The calibration curve was represented by the following linear regression equations: y = 284417x-136 (R^2=0.9999) (Fig. S2).

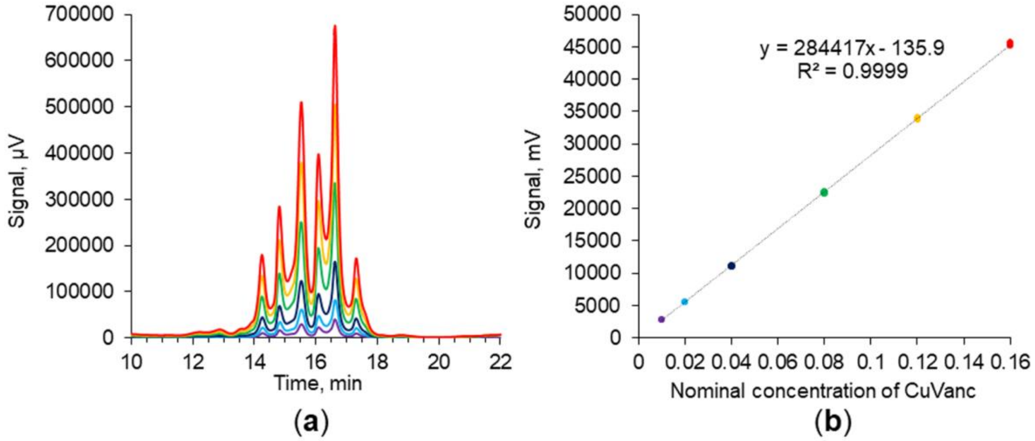

Figure S2. Chromatograms of calibration solution of crude NC (**a**) and calibration curve (**b**).

Additionally, repeatability of signals area of CuVanc NCs (nominal concentration 0.1) was measured in 5 replicates, RSD was 4.0%.

Validation of a capillary electrophoresis method for determination of Cu^{2+}

Stock solutions of $CuSO_4$ was prepared in water to achieve a concentration of 10 mM of Cu^{2+}. Calibration solutions were prepared by diluting the corresponding stock solutions in water to obtain a final concentration of 0.05, 0.10, 0.25, 0.50, 0.75, 1.00 and 2.50 mM. Quality control (QC) samples were prepared by diluting the corresponding stock solutions in water to achieve a final concentration of 0.05 (LLOQ), 0.15 (QCA), 1.25 (QCB), 1.875 (QCC) mM.

Background electrolyte consist of 1 mM CTAB, 8 mM EDTA, 50 mM acetate buffer solution (pH 5.5). A working voltage was - 15 kV. A working temperature was +20 °C. Spectrophotometric detection was performed at λ = 225 nm. A sample injection was hydrodynamic 10s × 50 mbar.

Calibration was carried out using 7 points. For each point, 3 measurements were made. The data were averaged and calibration curves calculated. Evaluated methods are linear between 0.05 – 2.500 mM for Cu^{2+}. The calibration curves were represented by the following linear regression equations: $y = 419.8x+7.7$ (R^2 = 0.9993) (Fig. S3a). Lower limit of quantification (LLOQ) of the method was 0.05 mM (Fig S3b). The limit of quantitation (LOQ) was determined based on a signal-to-noise ratio. The LOQ was defined as the lowest concentration of Cu^{2+} that produced a peak with an S/N ratio of 10:1. LOQ was 0.025 mM. The limit of detection was defined as a lowest concentration of the analyte that produced at S/N ratio of 3:1. The LOD was theoretically verified as 0.008 mM.

The average migration time for Cu^{2+} during the run was 4.86 ± 0.02 min (n = 20, p = 0.95), RSD = 0.4%, between 3 runs - 4.87 ± 0.04 min, RSD = 0.8%.

Selectivity was calculated in resolution (R_s) term based on separation of nearest peak. In blank solution (deionized water) no signal was observed for analytical run. In samples obtained during SPE nearest signal was observed at 3.4 min, R_s was 33.1 ± 1.2 (Fig S3b).

The precision of Cu^{2+} determination within a single run ranged from 0.4% to 2.0% and between runs from 1.9% to 11.2%, with an accuracy of 89.9% to 113.5% and 98.3% to 100.6%, respectively (Table S3).

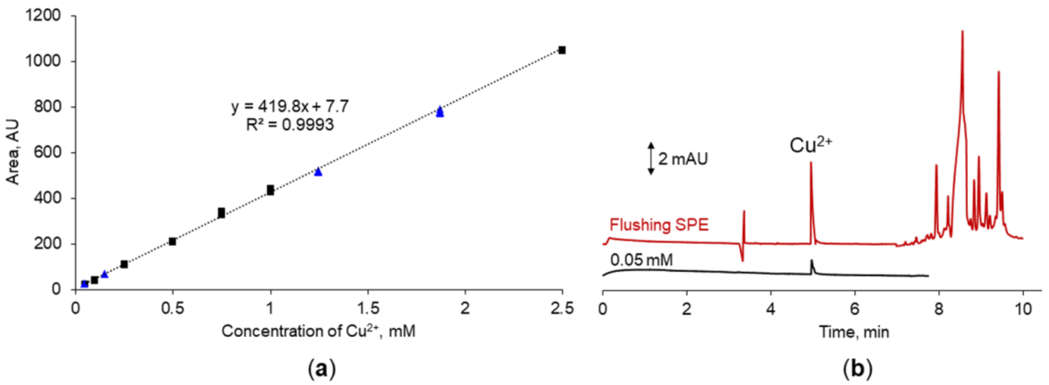

Figure S3. Calibration curve (**a**) with calibration (black squares) and quality control (blue triangles) samples and electropherograms (**b**) of LLOQ samples (black line) and collected fraction after flushing of the cartridge with 20% MeOH acidified by 0.1% formic acid (red line).

Table S3. Accuracy and precision of the method for Cu^{2+} determination.

				**LLOQ (0.05 mM)**			Average	RSD, %
Run 1	C, mM	0.046	0.047	0.046	0.045	0.046	0.046	1.0%
	Accuracy, %	92.6%	93.2%	91.4%	90.9%	92.0%	92.0%	
Run 2	C, mM	0.055	0.057	0.056	0.057	0.058	0.057	1.7%
	Accuracy, %	110.9%	114.9%	112.9%	113.3%	115.7%	113.5%	
Run 3	C, mM	0.045	0.044	0.046	0.046	0.044	0.045	2.0%
	Accuracy, %	90.4%	87.6%	91.3%	91.8%	88.6%	89.9%	
Inter-day Accuracy = 98.5%, Precision = 11.2%								
				**QCA (0.15 mM)**			Average	RSD, %
Run 1	C, mM	0.141	0.140	0.142	0.144	0.140	0.141	1.3%
	Accuracy, %	93.7%	93.5%	94.5%	96.1%	93.1%	94.2%	
Run 2	C, mM	0.158	0.158	0.157	0.159	0.158	0.158	0.4%
	Accuracy, %	105.5%	105.3%	104.9%	106.1%	105.5%	105.5%	
Run 3	C, mM	0.152	0.156	0.154	0.151	0.152	0.153	1.3%
	Accuracy, %	101.2%	103.9%	102.9%	100.7%	101.6%	102.1%	
Inter-day Accuracy = 100.6%, RSD = 4.9%								
				**QCB (1.25 mM)**			Average	RSD, %
Run 1	C, mM	1.201	1.213	1.225	1.204	1.211	1.211	0.7%
	Accuracy, %	96.1%	97.1%	98.0%	96.3%	96.9%	96.9%	
Run 2	C, mM	1.192	1.216	1.204	1.222	1.214	1.210	1.0%
	Accuracy, %	95.4%	97.3%	96.3%	97.8%	97.1%	96.8%	
Run 3	C, mM	1.281	1.269	1.242	1.260	1.279	1.266	1.3%
	Accuracy, %	102.5%	101.5%	99.3%	100.8%	102.3%	101.3%	
Inter-day Accuracy = 98.3%, RSD = 2.4%								
				**QCC (1.875 mM)**			Average	SD, %
Run 1	C, mM	1.840	1.828	1.823	1.868	1.835	1.839	1.0%
	Accuracy, %	98.1%	97.5%	97.2%	99.6%	97.9%	98.1%	

Run 2	C, mM	1.853	1.869	1.823	1.855	1.873	1.855	1.1%
	Accuracy, %	98.8%	99.7%	97.2%	98.9%	99.9%	98.9%	
Run 3	C, mM	1.929	1.881	1.911	1.924	1.917	1.912	1.0%
	Accuracy, %	102.9%	100.3%	101.9%	102.6%	102.3%	102.0%	
Interday Accuracy = 99.7%, RSD = 1.9%								

The average migration time for Cu^{2+} during the run were 4.86 ± 0.02 min (n = 20, p = 0.95), RSD = 0.4%, between 3 runs - 4.87±0.04 min, RSD = 0.8%.

References

[61] Guidance for Industry: Bioanalytical method validation. U.S. Department of Health and Human Services, Food and Drug Administration, Center for Drug Evolution and Research (CDER). U.S. Government Printing Office: Washington, DC, 2018.

[62] Guideline on bioanalytical method validation. European Medicines Agency. Committee for medicinal products for human use: London, 2011.
